# Supplementary material for: Intertemporal environmental efficiency assessment in China: A new network-based dynamic super-efficiency measure
Source: PLoS One. 2023 Aug 31;18(8):e0290896. doi: 10.1371/journal.pone.0290896 (PMC10470937; doi:10.1371/journal.pone.0290896)
Supplement: Attachment — Submitted filename: Responses.pdf [file pone.0290896.s005.pdf]

Responses to the Comments on our Paper

Intertemporal environmental efficiency assessment in China: a new network-based dynamic super-efficiency measure

Manuscript ID: PONE-D-23-17252

We'd like to express our heartfelt thanks to the editor for giving us an opportunity to revise the manuscript. We do appreciate it very much!

During the past several weeks, we have modified our manuscript carefully according to the reviewers' reports. All the comments raised by the two reviewers are answered. What follows are our detailed responses. To distinguish, we put their original comments in Italic in the following. Please see the following point-by-point responses.

Editor's Comments:

1. Please ensure that your manuscript meets PLOS ONE's style requirements, including those for file naming. The PLOS ONE style templates can be found at ...

Answer: We have downloaded the latex template of plos from the website, and have applied the article format according to the template requirements. We hope the present format of the manuscript can meet PLOS ONE's style requirements.

2. Thank you for stating the following in the Acknowledgments Section of your manuscript: "The work was supported by the National Natural Science Foundation of China [grant number 71971163]."

We note that you have provided funding information that is not currently declared in your Funding Statement. However, funding information should not appear in the Acknowledgments section or other areas of your manuscript. We will only publish funding information present in the Funding Statement section of the online submission form. Please remove any funding-related text from the manuscript and let us know how you would like to update your Funding Statement. ... Other authors received no specific funding for this work."

Please include your amended statements within your cover letter; we will change the online submission form on your behalf.

Answer: We have deleted the funding information from the Acknowledgments section of the revised manuscript.

3. We note that Figure 1 in your submission contain [map/satellite] images which may be copyrighted. ... We require you to either (a) present written permission from the copyright holder to publish these figures specifically under the CC BY 4.0 license, or (b) remove the figures from your submission

Answer: Figure 1 in the original manuscript is our original drawing and does not involve any copyright issues. We don't know why we have this issue. In order to comply with the journal's request, we removed this figure in the revised manuscript. We hope that this change will not affect readers' reading of this article. Please see the modification on page 4.

4. Please include captions for your Supporting Information files at the end of your manuscript, and update any in-text citations to match accordingly.

Answer: We have made changes in the revised manuscript. We hope that this revision can meet the requirements of the journal.

5. Please review your reference list to ensure that it is complete and correct. ...If you need to cite a retracted article, indicate the article's retracted status in the References list and also include a citation and full reference for the retraction notice.

Answer: We have checked our reference list. We ensure that it is complete and correct.

All the comments raised by the three reviewers are answered. Please see the following point-by-point responses.

Reviewer 1's Comments:

Abstract: The abstract should sequence the objectives, techniques, and concise results. Revise accordingly. Check the sequence of keywords.

Answer: We have revised the abstract according to this comment and the sequence of keywords has also been adjusted. Please see the revised abstract and keywords.

Introduction: I can see that the introduction section is lengthy. Exclude unnecessary information and be concise with the study's research problem, gaps, objectives, and innovative contribution if possible. Usually, at the end of the introduction section, the authors describe the innovations and route of the study. Revise accordingly to put the introduction section in the appropriate shape.

Answer: According to this comment, we greatly reduced the content of the introduction section, which briefly introduces the research problems, gaps, objectives, and innovative

contribution. Please see the revised introduction section from page 2 to page 3.

After combining the opinions of other reviewers, the penultimate paragraph of introduction describes the main innovations and routes of the study, and the last paragraph describes the structure of the article. Please see the last two paragraphs of the introduction section on page 3.

Methods: Proposed methods are accurate and innovative. Add more citations in the interpretation of the result section. Further, elaborate on the results in detail.

Answer: Combining this opinion with reviewer 2's comments, we significantly modified Section 2 and Section 3, mainly adding the explanation of the model. In the part of empirical results, we mainly added explanations for the selection of indicators, relevant literature citations, and comparisons with existing methods, deleted some unnecessary descriptions, and carried out further analysis of empirical results. I hope the current modification can meet the reviewers' requirements.

Conclusions and policy implications. Add limitations and future research ideas in the conclusion section. Give more practical implications of your proposed model.

Answer: We have added limitations and future research ideas in the revised conclusion section. More practical implications of the proposed model are also added in the first paragraph of the revised conclusion section. Please see the modification from page 17 to page 18.

Avoid grammatical and typo errors, and revise the manuscripts for these concerns.

Answer: We have carefully reviewed the manuscript many times and have corrected many grammatical and typos. I hope the revised manuscript can meet the reviewers' requirements.

Carefully check and revise the table and figure numbers in the manuscript.

Answer: In the revised manuscript, we have deleted 2 figures, and now there are 4 figures and 7 tables. We also corrected the quotes in the revised manuscript.

Reviewer 2's Comments:

1. The authors should enhance the clarity and organization of the manuscript by providing a more straightforward introduction that clearly states the research objectives, the significance of the research question, and the methodology employed. Additionally, it would be helpful to provide a brief overview of the structure of the paper to guide readers through the sections.

Answer: We have greatly reduced the content of the introduction section, which briefly introduced the research problems, gaps, objectives, and innovative contribution. The last paragraph of the introduction section has described the structure of the article. Please see the revised introduction section from page 2 to page 3.

2. The manuscript would benefit from a more detailed explanation of the network-based dynamic DEA model and the dynamic super-efficiency DEA model proposed by the authors. Including the mathematical formulations, constraints, and variables used in these models would assist readers in understanding the methodology and replicating the analysis.

Answer: Thanks for this comment! Although we also have explanations of variables and constraints in the original manuscript, the layout is messy. In the revised manuscript, we concentrated the description of the variables and constraints at the bottom of the models. Please see the modification from page 4 to page 9.

3. Provide a comprehensive description of the data sources used in the study, including information on the variables used to measure environmental efficiency and regional differences. Additionally, it would be beneficial to clarify how the DMUs were selected and if any data preprocessing techniques were applied.

Answer: According to this comment, we revised Table 2 to describe the data source. Please see the revised Table 2 on page 11.

In addition, it should be noted that we did not specifically select indicators to measure regional differences. We only selected capital, labor, and energy as inputs, polluting emissions as undesirable outputs, and GDP as desirable outputs according to the literature on environmental efficiency evaluation. It's just that in reality, the differences between different regions do exist, rather than the variables we chose to measure the differences.

Since we measure inter-provincial environmental efficiency, we definitely choose provinces as DMUs. Since DEA is a dimensionless technology, all the data are directly substituted into the models to calculate without data processing. Please see our description on pages 10 and 11.

4. The authors should present the results of the dynamic DEA and super-efficiency DEA models.

Answer: According to our method, the period efficiency scores are derived from these two models, so in order to avoid duplication, we only add the overall efficiency scores generated by these two models in Table 3. See the changes in Table 3 and the related

modification from page 11 to page 12.

5. The findings related to the regional differences in environmental performance should be summarized and discussed concisely and coherently. Provide clear explanations of the implications and policy recommendations that arise from the results.

Answer: Based on this comment and combined with the comments of other reviewers, we have significantly reduced the description of regional differences. The explanations of the results has been revised accordingly and a subsection has been added to present the policies. Please see the modification on pages 12, 13 and 17.

6. The manuscript would benefit from a discussion of the limitations of the proposed models and potential avenues for future research. Addressing these aspects would enhance the paper's contribution and help readers understand the broader context of the study.

Answer: Thanks for this comment! Please see our modification on page 18.

7. Give a section number to each section

Answer: The template for Plos one requires no numbers in each section.

8. Revise for typo mistakes

Answer: We have carefully reviewed the manuscript many times and have corrected many typos. I hope the revised manuscript can meet the requirements.

Reviewer 3's Comments:

1.The indicators selected should be supplemented by explanations as to why they were chosen for empirical research.

Answer: Thanks for this important comment! We have added the explanations about why the indicators are chosen in the revised manuscript. Please see the modification on page 11.

2. References to policy recommendations or management decisions are overly simplistic, and in-depth discussions are recommended.

Answer: According to this comment, we have added an subsection about policy recommendations. Please see the modification on page 17. And some in-depth discussions are provided on pages 15-17.

3. Please strengthen the discussion of differences between similar studies and those studied, highlighting the contribution of this study.

Answer: According to this comment, we do two main modifications:

(1) We highlight the contributions of this study on page 3.

(2) In empirical research, we compare the results generated by our method with those generated by similar studies. Please see the modification from page 11 to page 12.

4. Please add the limitations and prospects of this study.

Answer: We have added the descriptions about limitations and prospects of this study on page 17.

We do hope that our thorough modifications and the above detailed illustrations are good enough to fully satisfy reviewers' and the editor's requirements, and our revised paper can be accepted to publish in Plos one!

We are looking forward to your positive response!
